# Improved survival of older patients with advanced breast cancer due to an increase in systemic treatments: a population-based study

**DOI:** 10.1007/s10549-019-05356-z

**Published:** 2019-07-19

**Authors:** Nienke de Glas, Esther Bastiaannet, Anna de Boer, Sabine Siesling, Gerrit-Jan Liefers, Johanneke Portielje

**Affiliations:** 1 Department of Medical Oncology, Leiden University Medical Center, P.O. Box 9600, 2300 RC Leiden, The Netherlands; 2 Department of Surgery, Leiden University Medical Center, Leiden, The Netherlands; 3 Department of Research, Netherlands Comprehensive Cancer Organization, Utrecht, The Netherlands; 4 Department of Health Technology and Services Research, University of Twente, Enschede, The Netherlands

**Keywords:** Breast cancer, Survival, Chemotherapy, Geriatric oncology, Epidemiology

## Abstract

**Purpose:**

The number of older patients with breast cancer is rapidly increasing. A previous study showed that between 1990 and 2005, the survival of older patients with breast cancer did not improve in contrast to younger patients. In recent years, scientific evidence in the older age group has increased and specific guidelines for older women with breast cancer have been developed. The aim of this study was to assess changes in survival outcomes of older patients with breast cancer.

**Patients and methods:**

All patients with breast cancer between 2000 and 2017 were included from the Netherlands cancer registry. We assessed changes in treatments using logistic regression. We calculated changes in relative survival as proxy for breast cancer mortality, stratified by age and stage.

**Results:**

We included 239,992 patients. Relative survival improved for patients < 65 for all stages. In patients aged 65–75 years, relative survival did not improve in stage I–II but did improve in stage III breast cancer (RER 0.98, 95% CI 0.96–1.00, *p* = 0.046). Concurrently, prescription of systemic treatments increased. In patients > 75, relative survival did not improve in patients with stage I/II or stage III disease, nor did treatment strategies change.

**Conclusions:**

This study shows that relative survival of patients aged 65–75 years with advanced breast cancer has improved, and concurrently, prescription of systemic treatment increased. To improve survival of patients > 75 as well, future studies should focus on individualizing treatments based on concomitant comorbidity, geriatric parameters and the risk of competing mortality and toxicity of treatments.

## Introduction

In recent years, the number of older patients with breast cancer is strongly increasing in Western countries due to ageing of their populations [1]. Treatment of older patients with breast cancer is based on limited evidence, since older patients are often excluded from randomized clinical trials [2, 3]. In addition, it has been shown that older patients in clinical trials are not representative for the general older population, since they have less comorbidity, a better socio-economic status, more favourable tumour characteristics and a better survival [4]. Furthermore, treatments may be less effective in older patients due to shorter residual life expectancy, competing risks of dying of other causes than breast cancer and higher risks of toxicity. Older patients generally receive less-extensive locoregional and adjuvant treatment compared to their younger counterparts [5]. However, it is not always clear whether less-extensive treatment is justified. A previous study showed that in the Netherlands, survival of older patients with breast cancer did not improve between 1990 and 2005, in contrast to younger patients in which 5-year relative survival increased from 84 to 89% [6].

However, in the past years, with many studies specifically addressing older women with breast cancer, scientific evidence for this age group has increased. The International Society for Geriatric Oncology has provided a consensus guideline in 2007 that summarizes all the available evidence for this specific patient group [7]. The guideline was updated in 2012 [8], and many more studies and insights have become available since.

Therefore, the aim of this study was to assess if these new insights have resulted in an improvement in relative survival outcomes in a national population-based cohort of older patients with breast cancer after 2005, and to compare this with younger patients with breast cancer. In addition, the study aimed to provide an overview of changes in treatment strategies in different tumour stages in the past two decades per age group [5].

## Methods

Data from the Netherlands Cancer Registry were used. This registry contains detailed information on tumour, treatment and survival outcomes. All patients in the Netherlands who are diagnosed with cancer are included in the registry based on notification by the national pathology database (PALGA). The national hospital discharge databank, which receives discharge diagnoses of admitted patients from all Dutch hospitals, completes case ascertainment. Survival status is retrieved by linkage with the municipal population registries and was completed up to January 1st 2018.

For the current study, all female patients with invasive breast cancer (stage I–IV) who were diagnosed between 2000 and 2017 were included. Patients were divided into three groups based on age at diagnosis: < 65, 65–75 and > 75 years. If patients were diagnosed with bilateral breast cancer, the characteristics of the most aggressive tumour were used for the analyses. This was defined in the following order: the largest tumour, the highest grade, or being ER and PR negative. This method was also applied if patients presented with multifocal carcinoma’s. If patients had more than one new primary breast cancer, only the first incident breast cancer was used for the analyses. This means that patients were allowed in the study if they had more than 1 primary breast cancer, but the tumour characteristics were used from the first incident breast cancer. In situ breast cancers were not taken into account. If variables were missing, they were analysed in a “missing” category within the same variable.

The study was approved by the review board of the Netherlands cancer registry.

### Statistics

For all analyses, STATA version 13.0 was used. All tests were two-sided and a *p* value of < 0.05 was considered as statistically significant. First, descriptive statistics using Chi square tests were used to compare differences in tumour and treatment characteristics between age groups.

Second, we composed figures depicting the number of patients with incident breast cancer per age group.

Third, we assessed changes in treatment patterns (surgery, radiotherapy, endocrine therapy and chemotherapy) over time, stratified by age group and tumour stage (stage I–II, stage III and stage IV). We calculated coefficients for changes in treatment strategies per year to gain insight in patterns of treatment. For this, we used logistic regression and reported the calculated change displayed as coefficient with the corresponding *p* value.

Next, we calculated survival outcomes per age group. Survival status was available until December 31st, 2017. Since the Netherlands Cancer Registry does not provide information on causes of death, we calculated relative survival as a proxy for breast cancer-specific survival using the Ederer II method by linkage to national mortality files retrieved from the national bureau for statistics (Statistics Netherlands), matched by age, stage and year of diagnosis. This is a validated and frequently used method to estimate cancer-specific survival [9]. The excess risk of mortality is displayed as Relative Excess Risk (RER), which can be interpreted as increase of relative mortality compared to the background population per year. Again, we performed additional stratified analyses per tumour stage and age groups. Additional multivariable survival analyses were adjusted for relevant tumour characteristics (including TNM-stage, grade, hormone-receptor status and HER2 status) and, in a second step, treatment characteristics (surgery, radiotherapy and systemic treatment). To provide graphical information, we composed graphics that depict 5-year relative survival over time. However, all analyses (displayed as Relative Excess Risk of mortality (RER)) were not censored and include all available follow-up time.

## Results

### Patient characteristics

A total number of 239,992 patients were included from the Netherlands Cancer Registry. Patient characteristics are presented in Table 1. Tumour characteristics strongly differed between different age groups. Older patients presented with more advanced tumours (43.2% of patients < 65 had stage I disease compared to 26.4% of patients aged > 75, *p* < 0.001). Older patients received less surgical treatment (66.0% in patients > 75 vs 95.6% of patients aged < 65, *p* < 0.001), and less radiotherapy compared to younger patients (27.7% in patients > 75 vs 68.3% in patients < 65, *p* < 0.001). Chemotherapy was rarely prescribed in older patients (1.3% of patients > 75 received chemotherapy as monotherapy and 0.5% of patients received endocrine combined with chemotherapy), but the majority of patients in the oldest age group received endocrine therapy as monotherapy (63.8%).

**Table 1:** Patient characteristics

	< 65		65–74		≥ 75		*p* value
	*n*	(%)	*n*	(%)	*n*	(%)	
TNM-stage							
I	60,940	(43.2)	29,527	(54.4)	11,809	(26.4)	< 0.001
II	57,797	(41.0)	17,847	(32.9)	20,845	(46.6)	
III	14,425	(10.2)	3938	(7.3)	6169	(13.8)	
IV	6153	(4.4)	2252	(4.1)	3581	(8.0)	
Unknown	1667	(1.2)	701	(1.3)	2341	(5.2)	
Grade							
I	26,465	(18.8)	13,125	(24.2)	6126	(13.7)	< 0.001
II	54,359	(38.5)	23,639	(43.6)	14,972	(33.5)	
III	40,880	(29.0)	11,055	(20.4)	9001	(20.1)	
Unknown	19,278	(13.7)	6446	(11.8)	14,646	(32.7)	
Year of diagnosis							
2000–2004	36,390	(25.8)	12,171	(22.4)	12,004	(26.8)	< 0.001
2005–2009	39,655	(28.1)	13,503	(24.9)	12,052	(26.9)	
2010–2014	41,196	(29.2)	17,047	(31.4)	12,880	(28.8)	
2015–2017	23,741	(16.8)	11,544	(21.3)	7809	(17.5)	
ER/PR status							
ER/PR negative	19,493	(13.8)	5237	(9.7)	4435	(9.9)	< 0.001
ER and/or PR positive	90,531	(64.2)	36,773	(67.8)	28,471	(63.6)	
Unknown	30,958	(22.0)	12,255	(22.6)	11,839	(26.5)	
HER2-status							
Negative	79,652	(56.5)	32,704	(60.3)	21,861	(48.9)	< 0.001
Positive	15,215	(10.8)	3508	(6.5)	2678	(6.0)	
Unknown	46,115	(32.7)	18,053	(33.2)	20,206	(45.2)	
Surgical treatment							
No	6207	(4.4)	3023	(5.6)	15,222	(34.0)	< 0.001
Yes	134,775	(95.6)	51,242	(94.4)	29,523	(66.0)	
Radiotherapy							
No	44,755	(31.7)	18,440	(34.0)	32,345	(73.3)	< 0.001
Yes	96,227	(68.3)	35,825	(66.0)	12,400	(27.7)	
Systemic treatment							
None	42,687	(30.3)	25,205	(46.4)	15,417	(34.5)	< 0.001
Endocrine therapy	20,631	(14.6)	20,315	(37.4)	28,530	(63.8)	
Chemotherapy	27,218	(19.3)	3825	(7.0)	564	(1.3)	
Endocrine and chemotherapy	50,446	(35.8)	4920	(9.1)	234	(0.5)	

### Changes in treatment strategies

Treatment strategies over time are presented in Table 2. In patients with stage I–II breast cancer, the percentage of patients who received surgical treatment declined in all age groups, but this was most pronounced in the oldest age group (88.2% in 2000 to 67.9% in 2017*, p* < 0.001). Systemic therapy was increasingly prescribed in all age groups, but especially the prescription of endocrine therapy (either neoadjuvant or adjuvant or as primary treatment) strongly increased in the oldest patients (51.2% in 2000 to 63.3% in 2017, *p* < 0.001).

**Table 2:** Treatment characteristics over time, stratified by stage and age

			2000	2001	2002	2003	2004	2005	2006	2007	2008	2009	2010	2011	2012	2013	2014	2015	2016	2017	Coefficient	*p* value
Stage I–II	< 65	Surgery	99.5	99.5	99.6	99.5	99.4	99.5	99.5	99.3	99.3	99.4	99.2	99.2	99.2	99.3	99.2	99.0	99.1	96.9	− 0.09	< 0.001
		Radiotherapy	65.5	66	67.5	66.5	66.3	68.3	67.1	67.6	68	66.4	67.8	68.2	71.7	74.2	74.2	75	78.2	60.4	0.02	< 0.001
		Endocrine therapy	35.3	37.9	38.8	39.8	38.8	43.4	42.3	40.9	47.5	55.1	55.1	56.7	56.0	57.2	57.3	57.4	57.5	38.0	0.05	< 0.001
		Chemotherapy	43.4	44.2	47.4	44.4	45.8	46.9	48.0	47.6	53.1	59.1	56.5	57.2	57.4	56.2	55.6	54.8	45.4	41.5	0.02	< 0.001
	65–75	Surgery	99.3	99.1	99.2	98.9	98.6	98.8	98.8	98.6	98.8	98.8	98.3	98.1	98.9	98.2	98.6	98.6	98.0	97.2	− 0.05	< 0.001
		Radiotherapy	58.2	58.3	58.9	59.3	61.5	62.3	63.9	65.9	63.9	63.8	67.4	69.8	72.5	73.6	75.1	76.6	78.3	69.3	0.05	< 0.001
		Endocrine therapy	35.4	35.6	33.9	32.8	31.1	33.6	33.1	35.4	43.0	49.3	50.5	52.3	50.4	51.1	49.9	50.4	50.2	41.8	0.05	< 0.001
		Chemotherapy	5.1	6.6	6.2	5.6	5.6	5.1	5.1	5.1	10.1	15.3	15.0	15.0	17.3	17.9	19.4	18.8	16.2	14.4	0.09	< 0.001
	> 75	Surgery	88.2	89.5	87	83.9	81.2	78.5	77.1	75.3	74	73.9	70.9	68.9	68.6	67.6	67.4	69.1	68.4	67.9	− 0.07	< 0.001
		Radiotherapy	27.5	27.9	27.5	24.9	24.8	24.9	25.3	23.5	23.9	24.2	24	25.8	28.1	27.6	33.7	35.3	36.5	33.2	0.03	< 0.001
		Endocrine therapy	51.2	48.5	51.4	51.1	52.7	56.4	56.1	58.9	64.6	70.0	68.9	67.8	66.5	68.2	67.8	65.6	64.8	63.3	0.05	< 0.001
		Chemotherapy	0.3	0.3	0.5	0.5	0.3	0.3	0.5	0.3	0.6	0.3	0.8	0.5	0.5	0.8	0.7	1.3	1.4	1.4	0.09	< 0.001
Stage III	< 65	Surgery	89.3	90.1	89.3	96.8	96	97.7	96.6	97.4	97.3	98	97.9	98.6	95.9	97.2	96.6	64.4	95.7	93.9	0.03	0.002
		Radiotherapy	76.2	83.1	77.6	92.3	90.5	91.6	91.2	91.1	91.2	90.7	91	89.7	91.6	92.2	91.	88.7	89.3	43.6	− 0.06	< 0.001
		Endocrine therapy	52.7	59.0	55.5	70.1	64.7	71.3	68.6	69.3	71.4	71.7	71.5	76.6	74.1	75.9	78.3	75.9	77.9	35.4	0.02	0.001
		Chemotherapy	78.4	79.9	82.5	93.2	93.1	95.3	94.1	94.1	96.4	95.6	95.3	95.9	94.5	95.4	95.9	92.3	90.7	89.6	0.05	< 0.001
	65–75	Surgery	82.2	87.1	76.5	91.8	94.8	92.2	93.3	96.3	94.1	96.8	95.3	92	93.3	93.4	91.7	89.7	89	87.6	0.006	0.657
		Radiotherapy	71	80.2	68.2	82	85.3	84	82.1	87.1	86.6	90.8	83.5	82.1	84.3	83.6	83.3	82.1	84.3	51.7	− 0.02	0.009
		Endocrine therapy	58.9	66.3	71.8	73.7	72.4	77.9	74.6	70.9	76.1	78.1	73.2	73.4	77.6	77.9	76.7	76.3	72.4	58.2	0.004	0.626
		Chemotherapy	33.6	33.7	37.7	36.9	37.4	44.3	48.3	51.5	58.8	65.3	60.6	60.1	61.4	63.5	67.1	61.4	64.3	52.7	0.08	< 0.001
	> 75	Surgery	69.9	61.1	63.7	74.1	74.5	71.5	68.9	66.6	7	68.9	66.3	65.3	63	64.4	63.6	62	54.9	56.2	− 0.033	< 0.001
		Radiotherapy	39.3	39.6	42.5	54	54.9	50.8	49	49.5	49	50.8	45	46.6	46.9	44.7	47.7	48.1	40.5	34.1	− 0.014	0.008
		Endocrine therapy	76.6	74.9	76.9	77.5	76.1	73.8	73.6	75.0	78.4	75.4	75.7	75.5	78.2	73.2	78.3	69.1	70.6	72.2	− 0.01	0.049
		Chemotherapy	4.2	5.1	4.3	4.0	4.4	4.3	2.7	3.0	5.3	7.3	5.3	6.3	5.7	3.7	3.4	4.5	5.8	5.4	0.014	0.230
Stage IV	< 65	Surgery	38.6	31.2	36.4	31.3	36.2	36.6	32.2	24	33.7	33.1	27	30	28.3	29.3	29.2	31.3	30.6	25.4	− 0.02	< 0.001
		Radiotherapy	18.9	16.9	25.2	16	12.3	13.6	16.1	15	16.4	18.1	13.7	17.8	15.2	16.7	24.8	21.7	25.2	13.2	0.01	0.081
		Endocrine therapy	51.1	45.8	51.4	54.3	56.8	61.3	61.1	60.4	57.3	57.7	55.2	61.7	64.4	64.3	62.9	62.9	62.5	51.2	0.02	< 0.001
		Chemotherapy	59.6	59.4	61.3	56.6	54.5	53.7	58.7	52.1	56.7	61.6	59.7	57.6	59.9	56.9	56.3	64.4	58.2	54.9	0.001	0.844
	65–75	Surgery	31.8	27.1	25.9	24.5	27.7	25.6	19.5	19	18.8	28.9	14	17	20.6	27.3	26.7	15.9	18.4	20	− 0.03	0.004
		Radiotherapy	19.6	16.8	8.3	13.8	9.9	10.7	8.9	6	7.7	12.4	12.9	7.5	12.1	9.8	18.8	8.8	14.7	10.3	− 0.01	0.705
		Endocrine therapy	56.1	70.1	66.7	72.3	66.3	63.6	64.2	71.0	63.3	56.2	75.3	69.8	68.1	6.2	70.9	65.9	63.2	57.9	− 0.003	0.651
		Chemotherapy	35.5	28.9	26.8	26.6	37.6	34.7	32.5	32.0	36.8	37.2	26.9	32.1	32.6	39.2	32.1	28.2	27.9	32.4	− 0.003	0.750
	> 75	Surgery	24.9	23.5	12.4	18.6	19.3	17.1	10.4	12.3	11.7	13.5	15.6	10	8.7	15	15.7	11.2	9.8	8.2	− 0.05	< 0.001
		Radiotherapy	8.5	10.6	10.5	6.9	6.9	8.8	6	10.3	6.3	4.3	7	8.9	3.1	6.1	5.9	7	6.5	5.1	− 0.03	0.008
		Endocrine therapy	74.0	76.5	71.9	75.5	78.7	79.3	80.2	75.5	77.1	75.1	71.5	70.5	71.9	72.8	73.3	76.9	69.1	71.4	− 0.02	0.037
		Chemotherapy	4.5	6.7	5.7	6.9	5.9	5.7	5.5	9.7	11.2	9.2	8.1	8.9	5.6	6.6	11.4	9.1	10.6	8.2	0.03	0.005

In patients with stage III breast cancer, the percentage of patients who received surgical treatment only declined in patients aged 75 years or older (69.9% in 2000 to 56.2% in 2017, *p* < 0.001), and this was not accompanied by an increase in endocrine treatment (76.6% in 2000 to 72.2% in 2017, *p* = 0.049). Chemotherapy in the first year after diagnosis was prescribed in an increasing proportion of patients aged 65–75 years (33.6% in 2000 to 52.7 in 2017, *p* < 0.001) but not in the oldest patients, where chemotherapy is still rarely prescribed (less than 8% in all incidence years, *p* = 0.230).

In patients with stage IV breast cancer, the use of chemotherapy slightly increased in patients aged > 75 (4.5% in 2000 to 8.2% in 2017, *p* = 0.005) but not in patients aged 65–75 years. Again, the proportion of patients who received surgical treatment decreased in patients of all age groups.

### Relative survival outcomes

**Fig. 1:**
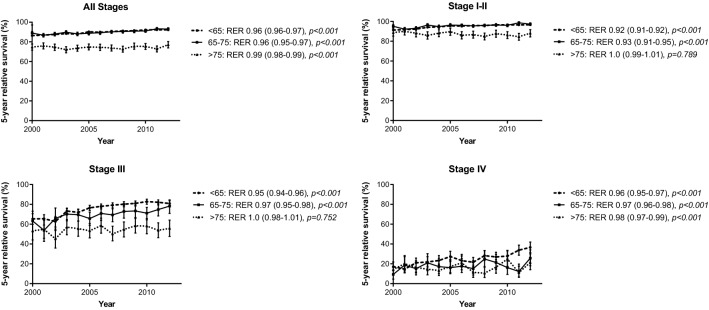
Relative survival over time per age-group, stratified by tumour stage

Median follow-up was 4.2 years (range 0–17.1 years). When combining patients of all stages, we showed that relative survival improved in all age groups (< 65: RER 0.95, 95% Confidence interval (CI) 0.95–0.96, *p* < 0.001, 65–75: RER 0.96, 95% CI 0.95–0.97, *p* < 0.001, > 75: RER 0.99, 95% CI 0.98–0.99, *p* < 0.001) (Table 3; Fig. 1). However, these changes were no longer present in patients aged 65–75 years and in patients > 75 years after adjustment for tumour characteristics (including tumour grade, stage, hormone-receptor status and HER2 status), while it remained statistically significant in the youngest age group.

**Table 3:** Relative survival stratified by stage and age

		RER	(95% CI)	*p* value
All stages	< 65			
	Year of diagnosis (crude)	0.95	(0.95–0.96)	< 0.001
	Year of diagnosis (adjusted for tumour characteristics*)	0.96	(0.96–0.97)	< 0.001
	Year of diagnosis (additionally adjusted for treatment**)	0.96	(0.96–0.97)	< 0.001
	65–75			
	Year of diagnosis (crude)	0.96	(0.95–0.97)	< 0.001
	Year of diagnosis (adjusted for tumour characteristics*)	0.99	(0.98–1.00)	0.217
	Year of diagnosis (additionally adjusted for treatment**)	1.00	(0.99–1.01)	0.698
	> 75			
	Year of diagnosis (crude)	0.99	(0.98–0.99)	0.001
	Year of diagnosis (adjusted for tumour characteristics*)	1.03	(1.02–1.03)	< 0.001
	Year of diagnosis (additionally adjusted for treatment**)	1.00	(0.99–1.01)	0.698
Stage I–II	< 65			
	Year of diagnosis (crude)	0.92	(0.91–0.92)	< 0.001
	Year of diagnosis (adjusted for tumour characteristics***)	0.93	(0.93–0.94)	< 0.001
	Year of diagnosis (additionally adjusted for treatment****)	0.93	(0.92–0.94)	< 0.001
	65–75			
	Year of diagnosis (crude)	0.93	(0.91–0.95)	< 0.001
	Year of diagnosis (adjusted for tumour characteristics***)	0.98	(0.97–1.00)	0.124
	Year of diagnosis (additionally adjusted for treatment****)	0.99	(0.97–1.00)	0.190
	> 75			
	Year of diagnosis (crude)	1.0	(0.99–1.01)	0.789
	Year of diagnosis (adjusted for tumour characteristics***)	1.07	(1.05–1.09)	< 0.001
	Year of diagnosis (additionally adjusted for treatment****)	1.0	(0.98–1.02)	0.738
Stage III	< 65			
	Year of diagnosis (crude)	0.95	(0.94–0.96)	< 0.001
	Year of diagnosis (adjusted for tumour characteristics***)	0.97	(0.96–0.98)	< 0.001
	Year of diagnosis (additionally adjusted for treatment****)	0.97	(0.96–0.98)	< 0.001
	65–75			
	Year of diagnosis (crude)	0.97	(0.95–0.98)	< 0.001
	Year of diagnosis (adjusted for tumour characteristics***)	0.98	(0.96–1.00)	0.046
	Year of diagnosis (additionally adjusted for treatment****)	0.99	(0.97–1.00)	0.159
	> 75			
	Year of diagnosis (crude)	1.0	(0.98–1.01)	0.752
	Year of diagnosis (adjusted for tumour characteristics***)	1.02	(1.00–1.04)	0.049
	Year of diagnosis (additionally adjusted for treatment****)	1.0	(0.99–1.02)	0.742
Stage IV	< 65			
	Year of diagnosis (crude)	0.96	(0.95–0.97)	< 0.001
	Year of diagnosis (adjusted for tumour characteristics***)	0.98	(0.97–0.99)	< 0.001
	Year of diagnosis (additionally adjusted for treatment****)	0.98	(0.97–0.99)	< 0.001
	65–75			
	Year of diagnosis (crude)	0.97	(0.96–0.98)	< 0.001
	Year of diagnosis (adjusted for tumour characteristics***)	1.00	(0.98–1.01)	0.554
	Year of diagnosis (additionally adjusted for treatment****)	1.00	(0.98–1.01)	0.609
	> 75			
	Year of diagnosis (crude)	0.98	(0.97–0.99)	< 0.001
	Year of diagnosis (adjusted for tumour characteristics***)	1.02	(1.00–1.03)	0.006
	Year of diagnosis (additionally adjusted for treatment****)	1.00	(0.99–1.01)	0.754

*Adjusted for grade, stage, HR status

**Adjusted for grade, stage, HR status, treatment (surgery, radiotherapy, systemic treatment)

***Adjusted for grade, HR status

****Adjusted for grade, HR status, treatment (surgery, radiotherapy, systemic treatment)

Stratified by stage, we observed an improvement of relative survival over time for patients in the youngest age group for all stages. In patients aged 65–75 years, relative survival improved in all stages in the univariate analyses (univariate RER 0.93, 95% CI 0.91–0.95, *p* < 0.001 for stage I–II disease, RER 0.97, 95% CI 0.95–0.98, *p* < 0.001 for stage III and RER 0.97, 95% CI 0.96–0.98, *p* < 0.001 for Stage IV). However, after adjustment for tumour characteristics, survival only improved in patients with stage III breast cancer (RER 0.98, 95% CI 0.96–1.00, *p* = 0.046). After additional adjustments for treatment (including surgery, chemotherapy, radiotherapy and endocrine therapy), the association was no longer statistically significant (RER 0.99, 95% CI 0.97–1.00, *p* = 0.159).

In patients aged 75 years or older, relative survival did not improve in patients with stage I/II or stage III disease. In stage IV disease, relative survival did improve (RER 0.98, 95% CI 0.97–0.99, *p* < 0.001), but after adjustment for tumour and treatment characteristics, this was no longer the case (RER 1.02, 95% CI 1.00–1.03, *p* = 0.754).

## Discussion

This study shows that in contrast with the previous studies, the relative survival of patients aged 65–75 years with advanced breast cancer has improved in the last decades, and concurrently, systemic treatment increased in this age group. However, no improvements were observed in the age group > 75 years.

The survival gain in patients aged 65–75 years in patients with stage III breast cancer is most likely explained by changes in systemic treatments, as demonstrated by the fact that the observed improvement was no longer statistically significant after adjustment for treatment. The most notable changes in treatment observed were increased administration of chemotherapy. The increase in chemotherapy occurred despite the fact that the Dutch breast cancer guidelines explicitly state that the additional value of adjuvant chemotherapy in older patients with an ER+ early breast cancer is low in older patients [10]. Up to 2017, the guideline even advised that patients aged 70 years or older should not receive chemotherapy [11]. As a result, the number of older patients with stage III breast cancer who receive chemotherapy is still very low compared to other European countries, as was previously published by our group [12]. It was shown that only 10% of patients over the age of 70 with stage III breast cancer in the Netherlands receive chemotherapy, compared to 35.2% in Belgium. Concurrently, survival outcome was better in Belgium, although not statistically significant. The percentage of patients with stage III disease who received systemic chemotherapy did increase from 33.6% in 2005 to 52.6% in 2017 (in patients aged 65–75 years), but it is still likely that there is a group of patients who are currently undertreated in this group. Hopefully, the currently ongoing French ASTER trial (NCT01564056) will aid in the evidence for treatment with adjuvant chemotherapy in older patients with breast cancer. The study randomized between adjuvant chemotherapy combined with endocrine therapy, versus endocrine therapy alone in older patients with high risk breast cancer (using a genomic prediction tool), results are expected in 2020.

The lack of survival gain in patients aged > 75 years is mostly in line with a previous analysis of the Netherlands cancer registry data on survival of women with a breast cancer diagnosis between 1990 and 2005 [6], and may be explained by several factors. First, we hypothesize that older patients with stage III breast cancer are currently undertreated in the Netherlands, as mentioned above. Possibly, the survival of patients aged > 75 would further improve by increasing the use of chemotherapy, especially in fit older patients with advanced breast cancer. On the other hand, a large majority of older patients in our cohort received endocrine therapy, even if they had stage I–II breast cancer. This might not be justified in all patients, since the risk of dying from other causes (so-called competing mortality) strongly increases with age and might have resulted in increased mortality from other causes, for example thrombotic or cardiovascular events [13, 14].

In addition, the lack of survival gain in the oldest age group might be explained by the increasing proportion of patients aged 75 years or older with stage I–III disease who did not receive primary surgery. Similarly, the survival of patients aged 65–75 years with stage I–II disease did not improve while the percentage of patients receiving primary surgery slightly declined. Previous clinical trials showed that tamoxifen alone was inferior to treatment with surgery with adjuvant tamoxifen in older patients with respect to progression-free survival and overall survival although the latter was not statistically significant in a Cochrane review summarizing the trials (HR 0.86, 95% CI 0.73–1.00, *p* = 0.06) [15]. However, these studies have never been performed with aromatase inhibitors which are somewhat more effective and currently the drug of choice in primary endocrine therapy. Finally, a possible explanation of the lack of survival gain in the oldest age groups is the influence of competing causes of death. It is well known that the risk of dying from other causes strongly increases with age [13, 16]. Therefore, a relatively small proportion of older patients with breast cancer who die, die from their breast cancer. Hypothetically, it is possible that this proportion is so small, that there is not much to gain from therapy improvements for the oldest age groups in terms of breast cancer survival. This makes it even more important to weigh benefits and risks of treatment, since the risk of complications and adverse events strongly increases with age and comorbidity [17–19].

Interestingly, the survival of older patients with stage IV breast cancer did improve with a concurrent small increase of systemic chemotherapy in the first year of treatment. The survival gain disappeared after adjustment for tumour characteristics, but this was probably related to the more comprehensive registration of tumour grade and ER/PR status in the most recent years, which caused an interaction. An alternative explanation is the increasing use of CDK4/6 inhibitors. Unfortunately, this is not yet registered in the cancer registry which means that we could not investigate this hypothesis.

Because many studies have focused on tailoring breast cancer treatment to the older patient during the last two decades, we expected to see an improvement in relative survival for this age category, as was previously observed in younger patient. However, the improvement was only observed in patients aged 65–75 years with advanced breast cancer, but not in patients aged > 75. To further improve overall and breast cancer-specific survival outcomes in all older patients, it is essential to individualize treatment by incorporating concomitant diseases and geriatric parameters in treatment decisions [20]. It has been showed in many previous studies that geriatric parameters such as gait speed, functional status, cognitive functioning and nutritional status are strongly predictive of survival [21, 22]. Furthermore, these factors can be used to estimate the risk of treatment toxicity [23]. Prediction tools for breast cancer survival such as Adjuvant! Online and PREDICT do not incorporate any geriatric parameters [24, 25]. Therefore, we are currently developing a new prediction tool (The PORTRET tool, which stands for “Prediction of Outcome and Toxicity in older patients with bREasT cancer”), that will incorporate tumour characteristics, comorbidity and geriatric predictive factors. The tool will not only predict breast cancer recurrence and competing mortality outcomes, but also the risk of treatment toxicity, quality of life and functional decline.

The main strengths of this study are the large sample size, as this is a national cancer registry database with well-registered data of all consecutive patients with breast cancer in the Netherlands. The use of relative survival is an additional strength of the study, since this outcome is not biased by misclassification of causes of death, which is a common problem in older patients [26].

Of course, this study has its limitations. First, we did not have any information on comorbidity status or geriatric parameters in this dataset, as these data are not registered by the Netherlands Cancer Registry. Second, no recurrence or cause of death were available, but the use of relative survival as an alternative method has been shown to be a valid alternative. In addition, information on systemic treatments were only available for the first year after diagnosis.

In conclusion, the relative survival of patients aged 65–75 years with advanced breast cancer has improved in the previous decades, most likely due to an increase in systemic chemotherapy in patients with stage III breast cancer. However, no improvements were observed in the age group > 75 years with stage I–III breast cancer. Future studies should focus on individualizing treatments based on concomitant comorbidity, geriatric parameters and the risk of competing mortality and toxicity of treatments.
